# Host Range Expansion and Dual Ecological Roles of an Invasive African Seed Predator on Native and Introduced Plants in Hawai‘i

**DOI:** 10.3390/plants14233620

**Published:** 2025-11-27

**Authors:** Mohsen M. Ramadan, Midori Tuda

**Affiliations:** 1 State of Hawaii Department of Agriculture, Division of Plant Industry, Plant Pest Control Branch, 1428 South King Street, Honolulu, HI 96814, USA; 2 Laboratory of Insect Natural Enemies, Faculty of Agriculture, Kyushu University, Fukuoka 819-0395, Japan; 3 Institute of Biological Control, Faculty of Agriculture, Kyushu University, Fukuoka 819-0395, Japan

**Keywords:** invasive species, conservation, Bruchidae, host plant range, seed germination, apparent competition, Fabaceae

## Abstract

Invasive seed predators can severely affect the reproduction of long-lived trees, especially when host range expansion occurs. The beetle *Specularius impressithorax* (Chrysomelidae: Bruchinae), native to Africa, has become established in Hawaiʻi where it attacks the endemic coral tree (*Erythrina sandwicensis*; Wiliwili). Here, we report the infestation of an African coral tree (*E. livingstoniana*) by this beetle and assess its performance and oviposition patterns on native and non-native hosts. Field surveys showed that eggs were aggregated on both hosts but more abundant on *E. sandwicensis* than on *E. livingstoniana*. Laboratory assays revealed no difference in larva-to-adult survival between the two hosts, although adults emerging from *E. sandwicensis* were larger. Choice tests indicated no oviposition preference between the two *Erythrina* species, despite the larger seed size of *E. sandwicensis*. To explore potential host range expansion, trials were run on economic legumes with varying phylogenetic distance from *Erythrina,* which showed oviposition on peanut (*Arachis hypogaea*) with low but successful survival (10.3%), while no development occurred on broad bean or pigeon pea. More *E. sandwicensis* seeds germinated when infested by a single early-stage larva (70% germination) than when uninfested (20%), suggesting that minimal seed predation may facilitate germination because previously reported greater damage induced by infestation through adulthood reduces germination. Our findings highlight the ecological flexibility of an invasive bruchine, its potential to exploit other Faboideae plants, and the dual role of seed predators as both threats and facilitators of seed germination. These results have implications for conservation of endemic coral trees and for understanding invasion dynamics of shared seed predators. Additionally, we examined non-botanical substrate filled with seed powder for oviposition and compiled global host records of *S. impressithorax* to contextualize its host range expansion.

## 1. Introduction

Shared insect predators can enhance plant diversity by suppressing relatively abundant plant species, thereby reducing competition among plants [1]. By contrast, they can also induce apparent competition among plant species by preferentially feeding on abundant plants, which indirectly reduces other host plants through the increased abundance of the predator’s offspring [2]. Invasive seed predators may severely affect the reproduction of long-lived trees, especially when host range expansion occurs. Seed predators of Fabaceae belong mainly to the subfamily Bruchinae (Coleoptera: Chrysomelidae) [3,4,5,6] alongside five other beetle families and a few Lepidoptera. Bruchines are specious in all continents except Australia and Antarctica [6]. The subfamily Bruchinae includes 1346 valid species, 84% of which attack Fabaceae hosts [3,4]. Most species are host specific at the genus or tribe level [5,6,7,8,9], and several specialist species are used as biological control agents of noxious plants [7,10].

Plants of the genus *Erythrina* (Fabaceae: Phaseoleae), commonly known as coral trees, are flowering plants of Gondwanan origin broadly distributed in tropical and subtropical regions worldwide [11]. Neill (1993) recognized 113 *Erythrina* species globally—70 Neotropical, 31 African, and 12 Asian or Oceania species [12]. Twenty-two *Erythrina* species are endangered or critically endangered (IUCN Red List [13]). Many species are used as ornamental street or park trees [14], and in tropical regions they serve as shade trees in coffee and cocoa plantations [15]. Their seeds contain erythrina alkaloids [16].

**Figure 1:**
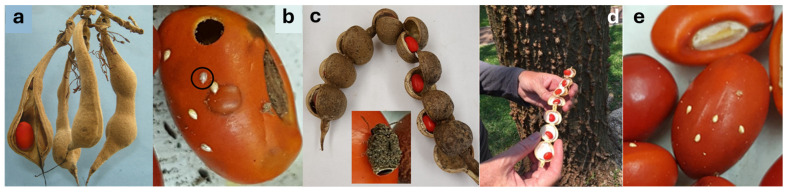
(**a**,**b**) *Erythrina sandwicensis* or Wiliwili, (**c**–**e**) *E. livingstoniana*. (**a**) Mature dehisced and indehiscent pods with seeds (11 February 2020). (**b**) A seed with hatched eggs, a dead embryo (circled, egg length = 0.77 mm), an adult emergence hole (top), and excised testa before adult emergence (center) (13 February 2020, O’ahu Island). (**c**) Mature dehisced pod with seeds; inset: adult *Specularius impressithorax*. (**d**) Tree trunk and seed pod. Ho’omaluhia Botanical Garden, O’ahu Island (11 February 2020). (**e**) Seeds with hatched eggs and one infertile egg.

Wiliwili, *Erythrina sandwicensis* O.Deg., a tree up to 15 m tall, with 1–3 red or yellow-orange seeds per pod [17] (Figure 1a), is the dominant species in lowland dry forests and the only endemic *Erythrina* species in the Hawai’ian Islands [11]. Traditionally, it was important to Native Hawai’ians for producing canoe outriggers, surfboards, and wreaths [18]. Today, Wiliwili is listed as Vulnerable by the IUCN [19], largely due to damage from the Erythrina gall wasp, *Quadrastichus erythrinae* Kim (Hymenoptera: Eulophidae) [20,21]. The internal seed-predatory bruchine beetle (feeding within seeds), *Specularius impressithorax* (Pic) (hereafter, SI; Figure 1c) (Coleoptera: Chrysomelidae: Bruchinae), native to Africa, arrived in the Hawai’ian Islands in 2001 [22,23]. SI females deposit eggs directly on seeds after pods dehisce but not on the pod surface [24] (Figure 1b); the hatching larvae bore into the seeds through the testa and develop into adulthood to emerge out through the excised exit hole (Figure 1b). Fully developed larvae likely damage radicles during the development, which eventually reduces germination success of *E. sandwicensis* (2.5% germination for seeds with one adult exit hole, and 0% for seeds with two or more adult exit holes, versus 68.5% germination for seeds with zero adult exit holes) [22]. As is typical for seed predators, SI likely entered Hawai’i in imported *Erythrina* seeds from Africa. Initial establishment probably occurred on the widely cultivated non-native host *E. variegata* [22]. Within three years of its first detection, SI caused a 77.4% mean seed crop loss in *E. sandwicensis* populations, posing a major threat. Adults feed on pollen and nectar.

*Erythrina livingstoniana* Baker, known as the aloe coral tree, grows up to 25 m tall [25] (Figure 1d). Its seed pod bears 6–8 red seeds (Figure 1c,d). The species is native to southeastern Africa—Malawi, Mozambique, Zambia, Zimbabwe, and South Africa—where it grows in woodlands. It is cultivated as an ornamental in tropical gardens and parks and is also used in traditional medicine for wound treatment [16].

This study compares the performance (survival of immature stages) and oviposition preference of SI on the newly found host *E. livingstoniana* (Figure 1e) and on its established host *E. sandwicensis*. To explore potential host range expansion, economic legumes (peanut, broad bean, and pigeon pea) with varying phylogenetic distance from *Erythrina* were tested for oviposition and larval development. Furthermore, we hypothesized that infestation by an early-instar larva may promote seed germination, contrary to previous findings that infestation through later stage (adult) inhibits germination [22]. Additionally, we tested whether non-botanical substrate (gelatine capsules) encapsulating seed powder can induce oviposition and complete larval development. We also compiled literature records of host associations to assess the global host range and invasion potential of this beetle.

## 2. Results

### 2.1. Plant Suitability and Seed Size Effects on Specularius impressithorax Egg Deposition

**Figure 2:**
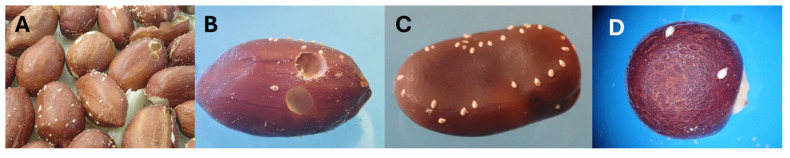
Rearing of *Specularius impressithorax* on economic non-*Erythrina* plants. (**A**) Peanut, *Arachis hypogaea*: oviposition and partial development; (**B**) peanut showing adult emergence holes; (**C**) broad bean, *Vicia faba*; (**D**) pigeon pea, *Cajanus cajan*.

**Figure 3:**
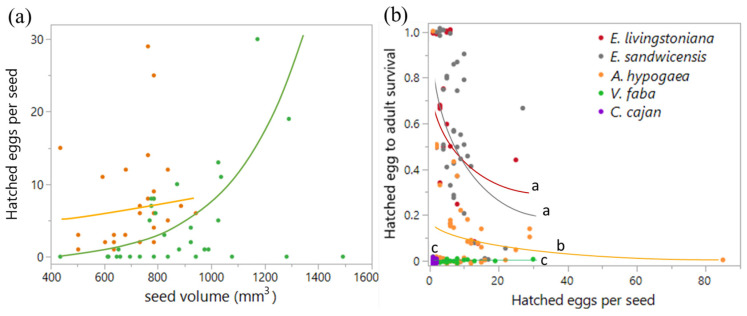
*Specularius impressithorax* oviposition and survival on different plant seeds. (**a**) Number of hatched eggs per seed increased with seed volume on broad bean *V. faba* (*p =* 0.004) but not on peanut *A. hypogaea* (*p* = 0.355). (**b**) Survival from hatched eggs to adulthood was different among plant species (*p* < 0.0001) and decreased with increasing hatched-egg density per seed (*p* < 0.0001) with plant species × hatched-egg density interaction (*p* < 0.0001). Shared letters indicate no significant difference. *C. cajan*: *Cajanus cajan* (pigeon pea). Line: model prediction. Jittered points in (**b**).

The onset of oviposition on raw peanuts *A. hypogaea* (seed volume = 724 ± 20 mm^3^, mean ± SE, *n* = 32) was delayed by one day. Development from hatched eggs to the last-instar larva proceeded successfully on *A. hypogaea* (survival = 0.199 ± 0.034, *n* = 50) (Figure 2A,B), while survival from hatched eggs to adulthood reached 0.103 ± 0.026 (*n* = 50) (Figure 3b). On *V. faba* (seed volume = 833 ± 35 mm^3^, *n* = 40), SI laid eggs on 18 seeds (45%) with a two-day delay, with up to 30 hatched eggs per seed (Figure 2C). Although larvae hatched, none developed further (Figure 3b). On *C. cajan*, eggs were laid on 22 seeds (44%), larvae hatched, but development did not proceed beyond the first instar (0.82 ± 0.10 eggs per seed, range 0–2, *n* = 50, Figure 2D and Figure 3b).

Hatched-egg density differed between plant species (Wald *χ^2^*_1_ = 22.58, *p* < 0.0001) and changed with seed volume (Wald *χ*^2^_1_ = 17.69, *p* < 0.0001) on peanuts and broad beans (Figure 3a). The plant species × seed volume interaction was marginally significant (Wald *χ*^2^_1_ = 3.62, *p* = 0.057). When analyzed separately, hatched egg density on *A. hypogaea* did not correlate with seed volume (Wald *χ*^2^_1_ = 0.86, *p* = 0.355), whereas on *V. faba* it increased significantly with seed volume (Wald *χ*^2^_1_ = 8.29, *p* = 0.004).

Survival from hatched eggs to adulthood differed among the five plant species (likelihood ratio (LR) *χ*^2^_4_ = 865.23, *p* < 0.0001). Survival decreased with hatched-egg density per seed (LR *χ*^2^_1_ = 401.10, *p* < 0.0001), and the interaction was significant (LR *χ*^2^_4_ = 458.31, *p* < 0.0001). Post hoc comparisons showed that survival was lower on non-hosts than on the *Erythrina* hosts (*E*. *livingstoniana* and *E. sandwicensis*), but among non-hosts, survival was higher on *A. hypogaea* than on *V. faba* or *C. cajan* (Figure 3b).

### 2.2. Egg Distribution, Egg and Larval Density Effects, and Infestation Capacity

More eggs were laid on Wiliwili (*E. sandwicensis*) than on *E. livingstoniana* in the field (Table 1): up to 33 eggs per *E. sandwicensis* seed and 26 per *E. livingstoniana* seed, comparable to a previous report of up to 38 eggs per field-collected *E. sandwicensis* seed [22]. Egg distributions on both *Erythrina* species followed negative-binomial rather than Poisson distributions, indicating aggregation (Table 1). Hatched-egg distributions were also negative-binomial for both *Erythrina* hosts and for peanuts and broad beans, but Poisson-distributed for pigeon pea (Figure A3).

**Figure 4:**
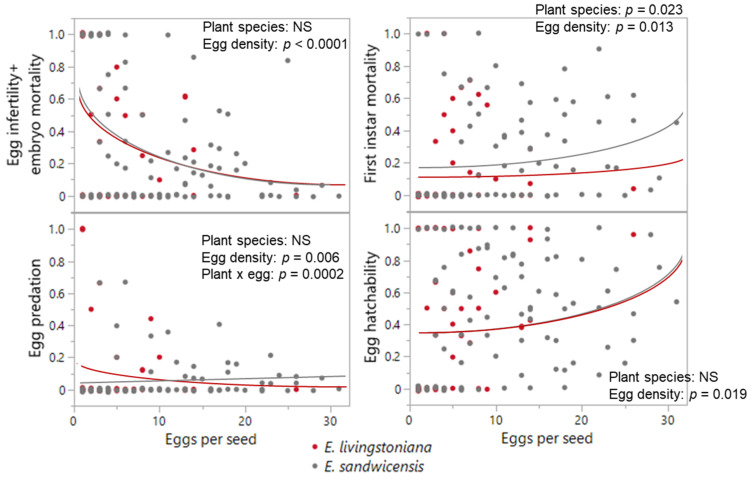
Effects of *Erythrina* species and egg density on the egg survival in *Specularius impressithorax.* No difference in mortality traits was found between *Erythrina livingstoniana* and *E. sandwicensis* (Wiliwili), except in the first instar mortality (higher on Wiliwili). Overall egg survival (hatchability) was positively correlated with egg density per seed. Line: model prediction. Data points are jittered along the y-axis.

**Figure 5:**
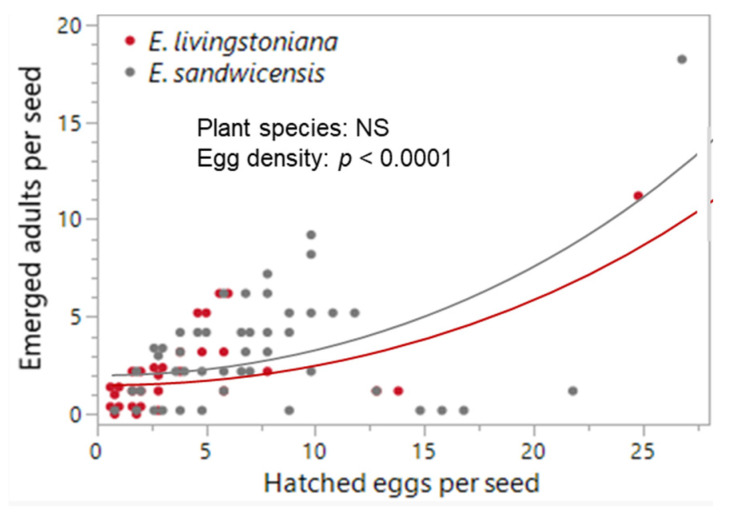
Effects of *Erythrina* plants and hatched-egg density on the number of emerged adults per seed in *Specularius impressithorax*. Number of emerged adults was not different between the two *Erythrina* species (*p* = 0.305) but increased with increasing hatched-egg density per seed (*p* < 0.0001). Line: model prediction. Data points are jittered.

**Figure A1:**
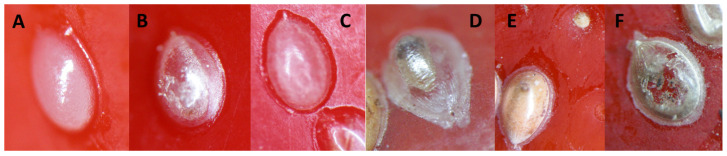
*Specularius impressithorax* egg categories on *E. livingstoniana* seeds: (**A**) newly deposited egg (oval; length: 0.77 ± 0.01 mm, *n* = 10), (**B**) infertile egg, (**C**) dead eggs with developed zygote, (**D**) dead first instar, and, (**E**) a hatched egg with first instar entered the seed (left) and a larval entry hole (top-right, Ø = 194.0 ± 6.7 µm, *n* = 10), (**F**) an egg predated upon with chewed chorion.

No difference was detected between the two *Erythrina* species in mortality traits or hatchability, except first-instar mortality, which was higher on *E. sandwicensis* (*p* = 0.023; Table 1, Figure 4 and Figure A1). Egg-density effects were significant for infertility + embryo mortality (negative; *p* < 0.0001), predation (host × egg density interaction *p* = 0.0002; negative only on *E. livingstoniana p* = 0.0002, on *E. sandwicensis p* = 0.266), first-instar mortality (positive; *p* = 0.013), and hatchability (positive; *p* < 0.0001) (Table 1, Figure 4 and Figure A1). Survival from hatched eggs to adults did not correlate with hatched-egg density per seed (*p* = 0.200; Table 1, Figure 3b). The number of emerged adults per seed did not differ between the two *Erythrina* hosts (Wald *χ*^2^_1_ = 1.05, *p* = 0.305) but increased with hatched-egg density (Wald *χ*^2^_1_ = 16.46, *p* < 0.0001) (Figure 5). Maximum adult emergence per seed was 18 for *E. sandwicensis* and 11 for *E*. *livingstoniana*.

Seed width and length were significantly greater and the volume was marginally greater in *E. sandwicensis* compared to *E. livingstoniana*, whereas height and weight did not differ (Table 1).

**Figure A2:**
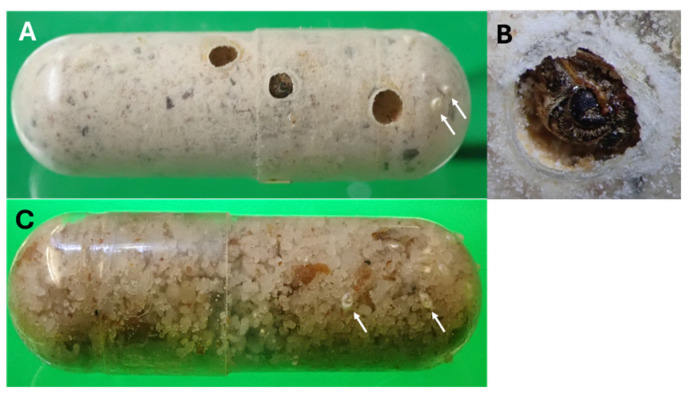
Gelatin capsules containing (**A**) ground *Erythrina variegata* seed powder with emergence holes, (**B**) adults failed to emerge from the hole, and (**C**) ground peanut (*Arachis hypogaea*) seed powder as an oviposition and growing medium for *Specularius impressithorax*. White arrows indicate laid eggs hatched on the surface of capsules.

**Figure A3:**
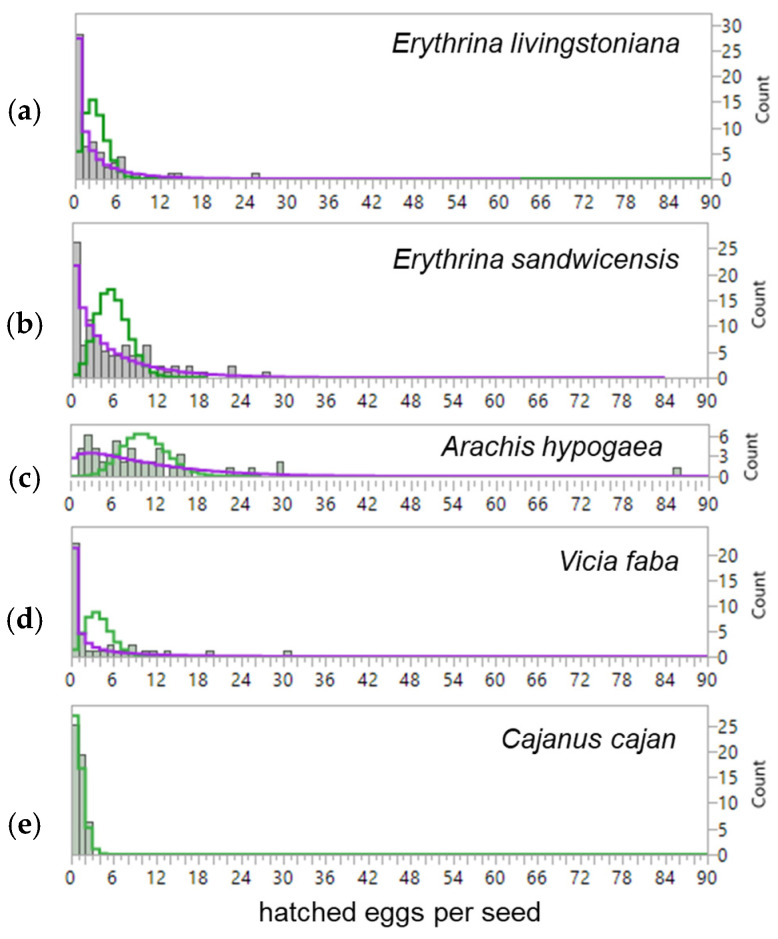
Distribution of number of hatched eggs per seed collected from the field for *Erythrina* and under the laboratory conditions for three economic Fabaceae species. Green line: Poisson distribution. Purple line: negative binomial (NB) distribution. (**a**) Poisson distribution, *χ*^2^ = 301.21, *n* = 60, *p* < 0.0001; NB distribution, *χ*^2^ = 2.74, *n* = 60, *p* = 0.434. (**b**) Poisson distribution, *χ*^2^ = 217.01, *n* = 98, *p* < 0.0001; NB distribution, *χ*^2^ = 8.22, *n* = 98, *p* = 0.412. (**c**) Poisson distribution, *χ*^2^ = 65.74, df = 5, *n* = 50, *p* < 0.0001; NB distribution, *χ*^2^ = 3.74, *df* = 5, *p* = 0.588. (**d**) Poisson distribution, *χ*^2^ = 102.95, df = 4, *n* = 40, *p* < 0.0001; NB distribution, *χ*^2^ = 1.58, df = 1, *n* = 40, *p* = 0.208. (**e**) Poisson distribution, *χ*^2^ = 0.59, df = 1, *n* = 50, *p* = 0.442; NB distribution failed to be fitted.

Females oviposited on empty gelatin capsules. Larvae completed development in capsules filled with *E. variegata* seed powder and adults emerged (*n* = 2) (Figure A2A,B), but larvae attempting to develop in capsules filled with peanut powder died after burrowing (*n* = 2; Figure A2C).

### 2.3. Body Size

**Figure 6:**
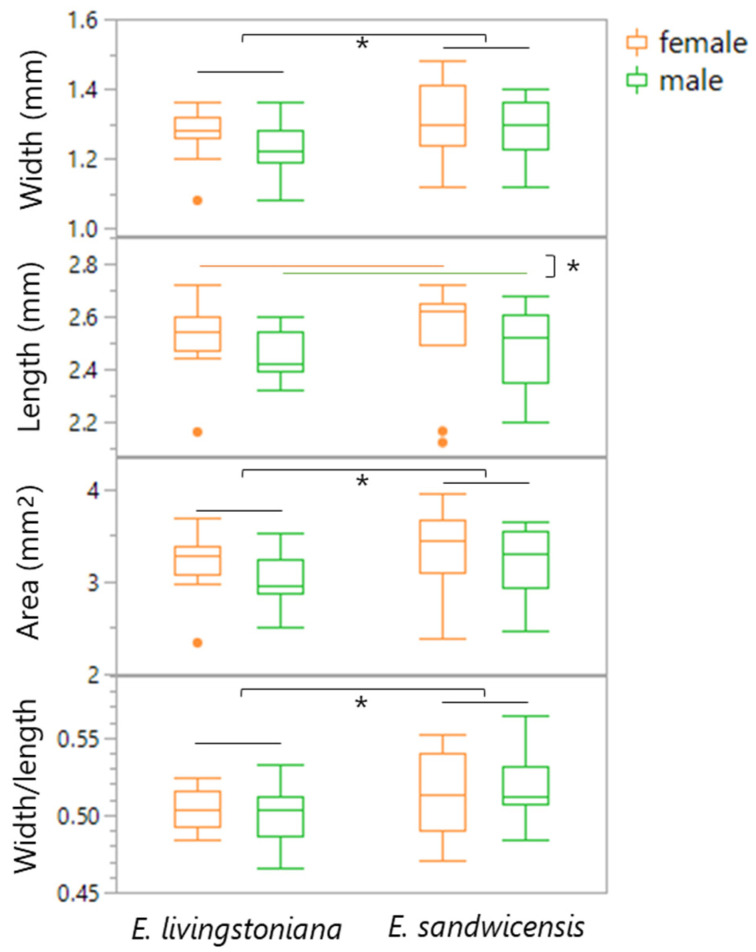
Box plots of adult morphological traits (elytron width, length, area, and width/length ratio) by sex of *Specularius impressithorax* emerging from *Erythrina livingstoniana* and *E. sandwicensis* (Wiliwili). Elytral width, area, and width/length ratio were different between rearing plant species, while elytral length was different between sexes, but not between rearing plants. *: *p* < 0.05.

Elytron width, elytron area, and width/length ratio were significantly larger in beetles reared on *E. sandwicensis* than on *E. livingstoniana*, with no effect of sex (Table 2, Figure 6). Elytron length did not differ between host plants but was significantly greater in females than in males.

### 2.4. Choice Experiment

One replicate yielded no oviposition and was removed. No difference occurred in egg number between *E. livingstoniana* and *E. sandwicensis* (*t* = 0.23, *df* = 4, *p* = 0.829).

### 2.5. Seed Germination

**Figure 7:**
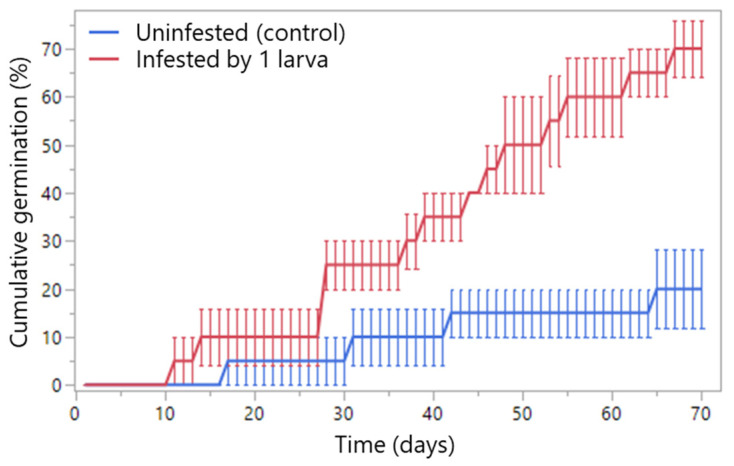
Cumulative germination (%) of infested (by a single early-stage larva) and uninfested Wiliwili seeds (*Erythrina sandwicensis*) over time (mean ± SE).

Time to germination differed significantly between treatments (LR *χ*^2^_1_ = 12.52, *p* = 0.0004), with infested seeds (by single early-stage larva) germinating earlier than uninfested controls (Figure 7). Replicate effects were non-significant (LR *χ*^2^_6_ = 4.23, *p* = 0.646). Final cumulative germination (day 70) reached 70.0 ± 5.77% for infested seeds, which was higher than 20.0 ± 8.16% for uninfested seeds (*F*_1,6_ = 25.00, *p* = 0.0025). All larvae died at the second instar, probably due to drowning.

### 2.6. Literature Review of Native Host Plant Range

**Figure A4:**
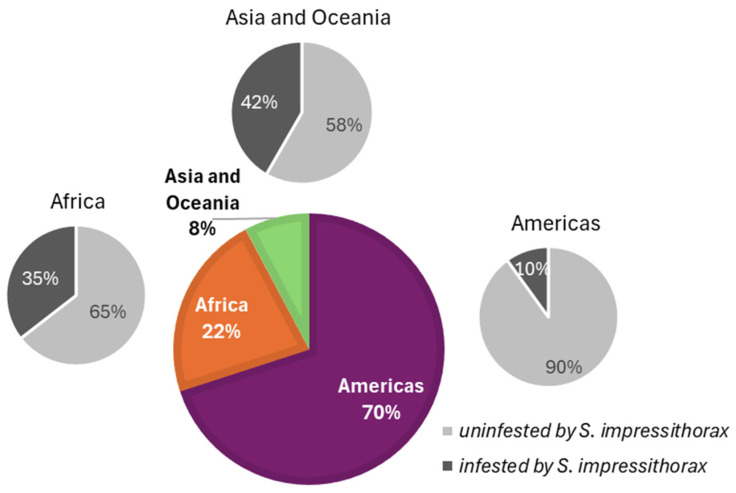
World *Erythrina* species distribution and utilization by *Specularius impressithorax*.

A literature review identified 22 *Erythrina* species used by SI (Table A1): 11 African/pantropical, 7 Neotropical, 1 native Hawaiian (*E. sandwicensis*), 2 from Indonesia/Philippines (*E. eudiphylla*, and *E. microcarpa*), and 1 from Australia (*E. sykesii*). The proportion of native *Erythrina* species utilized by SI was high in Asia/Oceania (41.7%) and Africa (35.5%), and low in the Americas (10.0%) (Figure A4). Non-*Erythrina* Fabaceae hosts have also been reported: the wooly wild bean *Strophostyles sarmentosa* (eastern North America) [26] and Calabar bean *Physostigma mesoponticum* (Angola) [27], both in Phaseoleae.

## 3. Discussion

*Specularius impressithorax* (SI), an African internal seed predator, is known to infest native Hawaiian *E. sandwicensis* and seven introduced *Erythrina* species in Hawai’i (Table A1). By comparing *E. livingstoniana* (native to southeastern Africa) with *E. sandwicensis*, we evaluated field infestation, laboratory survival, and host preference. Beetles reared from *E. livingstoniana* were smaller than those from *E. sandwicensis*. SI is best described as oligophagous: specialized on *Erythrina* but capable of opportunistic oviposition on non-host substrates. It develops in mature dry seeds, either still in dehisced pods on the plant or in fallen seeds. The use of dry mature seeds is likely an adaptation to arid native environments [28].

SI readily oviposited on non-host seeds and even inert objects (e.g., gelatin capsules), consistent with previous observations of oviposition on plastic beads [22]. The smooth surface and curvature may elicit oviposition as in other bruchine beetles [29,30]. Its ability to complete development on peanuts (*A. hypogaea*, tribe Dalbergieae), but not on broad bean (*V. faba*, tribe Fabeae) or pigeon pea (*C. cajan*, tribe Phaseoleae), indicates that factors beyond phylogenetic relatedness among plants influence the beetle’s developmental success. Ecologically, peanuts are inaccessible because they develop underground, consistent with their absence from natural host records (Table A1). SI’s specialization on *Erythrina* likely reflects larval digestive limitation against unadapted toxic chemicals such as alkaloids present in other Fabaceae [31,32]. *Erythrina* seeds disperse near maternal trees, producing dense local seed banks vulnerable to attack by multivoltine bruchines [31].

Our work provides the first confirmed record of SI infestation on *E. livingstoniana* [26,33]. Despite similar oviposition preference and immature survival between the two hosts and *E. livingstoniana* being native to SI’s range, *E. sandwicensis* received more SI eggs in the field. Several explanations are plausible. (1) Seed size [34]: *E. sandwicensis* seeds are marginally larger, which can promote oviposition, although the laboratory choice test did not support a strong preference. (2) Seed quality: Larger adult body size when reared on *E. sandwicensis* may indicate higher larval suitability for development. (3) Resource abundance [35]: On O’ahu, *E. sandwicensis* is substantially more abundant (131 trees) than *E. livingstoniana* (four cultivated trees) (botanical gardens; N.F. Hoffman, pers. comm.), offering more frequent oviposition opportunities for a multivoltine species. (4) Phenology: *E. sandwicensis* exhibits variable flowering and seed-set periods among trees [36], whereas *E. livingstoniana* flowers within a narrower window (our observations), reducing temporal availability.

Egg survival (hatchability) increased with egg density on both two *Erythrina*, although high larval density reduced survival to adulthood. Nevertheless, adult emergence often remained high, suggesting selective pressure favoring egg aggregation. Egg aggregation behavior is observed in other bruchines such as *Zabrotes subfasciatus* and *Acanthoscelides obtectus* that utilize *Phaseolus* seeds [37,38]. Hard, large seeds such as those of *Erythrina* and *Phaseolus* may favor group foraging by endophagous larvae.

Infestation by a single early-instar larva increased *E. sandwicensis* germination (from 20% of uninfested control to 70%), probably by enhancing water permeability via larval entry holes [6,39]. Germination success depends on the developmental stage of the seed predator: late-stage larval feeding or adult emergence suppresses germination, with only 2.5% germination after an adult exit hole compared to >68% of undamaged seeds [22] (or 73% [40]). The low germination of unin-fested controls (20%) in the present study likely reflects prolonged cold storage. Effects of seed predators on germination vary widely across plant taxa, seed sizes, and seed-predator densities [6,41,42,43,44], as observed for the bruchine *Acanthoscelides macrophthalmus* on *Leucaena leucocephala* seeds [42]. Higher germination percentage (66.6%) was recorded for *E. americana* seeds with a single SI adult emergence hole [45]. Furthermore, plant–insect coevolutionary history, interactions with mammalian seed predators, water availability, and environmental factors that aid seed scarification (e.g., forest fire) would all determine whether insect seed predation enhances germination [6,41,46,47,48]. Hawaii’s February rains may amplify early-damage-induced germination.

Use of introduced *Erythrina* species (e.g., *E. variegata* and *E. livingstoniana*) may elevate SI abundance, indirectly increasing seed predation on the endemic *E. sandwicensis* via apparent competition induced by shared predation [2]. Similar patterns occur globally in other invaded ecosystems: introduced hosts can subsidize populations of seed predators that then spill over onto native plants (e.g., scolytine beetles on endemic and non-native palms in Mediterranean coastal systems [49]; Neotropical *A*. *macrophthalmus* expanding from its native host *L. leucocephala* to the introduced *Falcataria moluccana* in Taiwan [50]; *Megabruchidius dorsalis* expanding from native *Gleditsia* species to introduced North American *Gleditsia triacanthos* and *Gymnocladus dioica* in Europe [51]. Collectively, such cross-host predator spillover highlights the potential for altered community composition to reshape native plant recruitment.

## 4. Materials and Methods

All experiments were conducted under laboratory conditions of 26 ± 1 °C during the day and 24 ± 1 °C at night, 60–80% RH, and 12 L:12 D h photocycle (under fluorescent and natural light).

### 4.1. Field Collection of Erythrina and Laboratory Rearing

Seeds of *Erythrina livingstoniana* and Wiliwili (*E. sandwicensis*) were collected at Ho’omaluhia Botanical Garden, Kaneohe, O’ahu Island, Hawai’i on 11 February 2020 (21°22′55.32″ N, 157°48′03.47″ W, elevation 96.9 m). Fallen ripe pods and seeds dropped from pods onto grass were collected randomly. Infestation was observed in fallen open pods, pods still attached to trees, and dropped seeds.

The number of hatched eggs and dead eggs or larvae (still within eggs) per seed due to infertility, predation, embryo death, or first-instar larval death was recorded (Figure A1), as were the numbers of emerged adults per seed. Observations continued until all adults emerged. These monitoring procedures were conducted under the laboratory conditions described above.

Width and length of the right elytron of emerged adults were measured post-mortem for 10 females and 10 males from each *Erythrina* species, using a calibrated ocular micrometer on a Leica 125 microscope. Sex of SI was determined both by antennal morphology (males possess stouter and more expanded serrate funicles) and confirmed by dissection for genital observation [24,26].

### 4.2. Host Plant Range, Egg and Larval Density Effects, and Seed Size

Three non-*Erythrina* economic legumes, peanut *Arachis hypogaea* L. (Fabaceae: Dalbergieae) (78 seeds), *Vicia faba* L. (Fabaceae: Fabeae) (40 seeds), and pigeon pea *Cajanus cajan* (L.) Huth (Fabaceae: Phaseoleae) (50 seeds), were tested as potential hosts. They were each exposed to 40, 20, and 20 SI adults, respectively, for oviposition and development. Dead adults were removed, and hatched eggs were counted. Seeds were held for 90 days from initial exposure to record adult emergence. When no adults emerged, seeds bearing hatched eggs were dissected to determine developmental stages of dead individuals.

Seed width, length, and height of both *Erythrina* species, and of *A. hypogaea* and *V. faba* were measured to the nearest 0.5 mm. Because seed size of *C. cajan* was relatively uniform, we did not measure it. Seed volume was estimated as

(1) $$V=\frac{4}{3}\pi \;\text{(width/2)}\cdot \text{(height/2)}\cdot \text{(length/2)}$$

### 4.3. Development Using Seed Powder

To test whether oviposition could be elicited by non-botanical material encapsulating seed material, powdered seeds of *Erythrina variegata* and *A. hypogaea*, prepared using an electric grinder, were placed into gelatin capsules. Each capsule was then exposed to adult SI for potential oviposition.

### 4.4. Choice Experiment

Oviposition choice between the two *Erythrina* hosts was tested using three seeds each of *E. livingstoniana* and *E. sandwicensis* placed on opposite sides of a Petri dish (9 cm diameter, 1.5 cm height) within a smaller dish (4 cm diameter, 0.6 cm height) each. Three unmated females and two unmated males (6 days old) reared on *E. variegata*—therefore previously unexposed to both test species—were confined together in a plastic cylinder (8 cm height, 2 cm diameter) for 24 h to allow mating. Only females were subsequently introduced to the Petri dish and allowed to oviposit for 48 h. Eggs were then counted. The test was replicated six times.

### 4.5. Seed Germination

Mature, undamaged Wiliwili seeds (collected from Koko Crater Botanical Garden, O’ahu in December 2008 and stored refrigerated) were exposed to five SI adults for 2 h in April 2025. Seeds bearing one egg were transferred to a Petri dish and incubated for 7 d to allow larval development under laboratory conditions (26 ± 1 °C light phase, 24 ± 1 °C dark phase, 12 L:12 D). Equal numbers (five) of infested or uninfested seeds were evenly spaced and allowed to germinate in between two filter papers moistened with distilled water in a Petri dish (9 cm diameter, 1.5 cm height) with four replications per treatment (20 seeds per treatment and eight dishes total). Dishes were enclosed in a polyethylene bag to minimize evaporation and kept under the same laboratory conditions. Germination was scored when the radicle had emerged ≥ 3 mm. Germination was recorded every 24 h for 70 d. Infested seeds were dissected at the end of the experiment to confirm larval death and developmental stages.

All procedures complied with relevant U.S. guidelines and regulations.

### 4.6. Literature Search for Host Plant Associations

A literature survey was conducted to compile host plant records of SI from global field observation and percentage utilization by SI was calculated.

### 4.7. Statistical Analysis and Vouchers

A generalized linear model (GLM) with a negative-binomial distribution and log-link function was applied to hatched eggs per seed, with plant species and seed volume, and their interaction effect as explanatory variables. A GLM with an exponential distribution and reciprocal link function was used for survival from hatched egg to emerged adults, with plant species, hatched eggs per seed, and their interaction effect as an explanatory variable. Likewise, GLMs with an exponential distribution and reciprocal link were used to examine the effects of host plant (two *Erythrina*), egg density, and their interaction effect on egg mortality (infertility + embryo mortality, egg predation, and first-instar larval mortality), egg hatchability (1—egg mortality), and survival from hatched eggs to adults. A GLM with a negative-binomial distribution and log-link function was applied to compare egg numbers per seed between the two host *Erythrina* species. A GLM with a negative-binomial distribution and log-link function was applied to emerged adults per seed, with plant species (two *Erythrina*) and hatched eggs per seed, and their interaction as an explanatory variable. A nonsignificant interaction effect was excluded from all models.

Seed size and mass between the two *Erythrina* species were compared by one-way ANOVA. Nonparametric one-sided Wilcoxon tests (with normal distribution approximation) were applied to assess effects of host plant and sex on elytron traits (width, length, area (=width × length), and width/length ratio) of emerged adults. A paired *t*-test was used to compare egg numbers in the choice experiment. Egg (on the two *Erythrina* hosts) and hatched-egg (on all five plants) distributions were fitted to Poisson and negative-binomial distributions. Time to germination with censoring was analyzed using a parametric survival analysis with a best-fit Weibull distribution (among Weibull, log-normal, exponential, Frechet and log-logistic distributions) with treatment and replicate nested within treatment. All statistical analyses were performed using JMP18.2.1 (SAS Institute, Cary, NC, USA). Voucher specimens are deposited in the insect collection of the Hawaii Department of Agriculture, Honolulu, Hawai’i.

## 5. Conclusions

The invasive African bruchine *Specularius impressithorax* continues to threaten native *Erythrina* populations in Hawai‘i through intensive seed predation and potential host range expansion. Our discovery of its successful development on the African *Erythrina livingstoniana* underscores its ecological flexibility and the risk of apparent competition across co-occurring *Erythrina* species. Beyond reporting a new host record, this study emphasizes the dual ecological role of seed predators: while the full development of beetle poses a threat to plant regeneration through extensive seed damage, early-stage larval infestation may facilitate germination by enhancing water absorption via larval entry holes, with minimal harm to the seed. Future work on host–parasitoid interactions [52] and chemical ecology [53,54] will be key to managing this invasive seed predator and conserving the native *Erythrina* flora.

## Figures and Tables

**Table 1:** *Specularius impressithorax* field egg infestation per seed of *Erythrina livingstoniana* and *Erythrina sandwicensis* (Wiliwili) (February 2020, O’ahu), and consequent laboratory rearing results. *n*: number of seeds. Unless otherwise noted, there was no significant plant × egg density interaction, which was then excluded from a model. *p*-values < 0.05 are bolded. 0.05 < *p* < 0.06 are italicized. Goodness of fit tests on egg distributions.

Parameter/Seed	*E. livingstoniana*		*E. sandwicensis*							
	*n*	Mean ± SE	*n*	Mean ± SE	Host Plant			Egg Density per Seed		
					*χ^2^*	*df*	*p*	*χ^2^*	*df*	*p*
In the field:										
eggs per collected seed	164	1.67 ± 0.28	103	9.92 ± 0.77	101.2	1	**<0.0001**	-	-	-
In the lab:										
eggs (excluding seeds with 0 egg)	58	4.59 ± 0.62	98	10.46 ± 0.77	36.12	1	**<0.0001**	-	-	-
egg infertility or embryo mortality	58	0.401 ± 0.058	98	0.271 ± 0.037	0.08	1	0.773	39.82	1	**<0.0001**
egg predation	58	0.091 ± 0.033	98	0.049 ± 0.013	0.02	1	0.884	7.62	1	**0.006** ^&^
first-instar larval mortality	58	0.127 ± 0.034	98	0.227 ± 0.031	5.14	1	**0.** **023**	6.11	1	**0.013**
egg hatchability	58	0.381 ± 0.054	98	0.453 ± 0.038	0.00	1	0.961	5.47	1	**0.0** **19**
					**Host plant**			**Hatched egg density per seed**		
survival from hatched eggs to adults	31	0.506 ± 0.071	47	0.464 ± 0.051	0.003	1	0.956	1.64	1	0.200
Seed size (cm), volume (mm^3^) and weight (g):										
Width	19	0.81 ± 0.013	20	0.86 ± 0.019	4.65	1, 37	**0.** **038**			
Length	19	1.37 ± 0.013	20	1.47 ± 0.037	5.44	1, 37	**0.** **025**			
Height	19	0.85 ± 0.011	20	0.85 ± 0.018	0.06	1, 37	0.809			
Volume (estimated)	19	498 ± 13	20	571 ± 34	3.80	1, 37	*0.059*			
Weight	19	0.649 ± 0.013	20	0.652 ± 0.035	0.004	1, 37	0.947			
**Egg distribution**	***n***	**Deviation**	***n***	**Deviation**						
Poisson	164	*χ*^2^ = 1242.7, *p* < 0.0001	103	*χ*^2^ = 622.6, *p* < 0.0001						
Negative binomial		*χ*^2^ = 139.1, *p* = 0.913		*χ*^2^ = 86.4, *p* = 0.866						

^&^: marginally significant interaction between host plant and egg density per seed; *χ*^2^_1_ = 14.17, *p* = 0.0002

**Table 2:** Elytron size of *Specularius impressithorax* reared on *Erythrina livingstoniana* and *E. sandwicensis* (Wiliwili) in the laboratory environment. Significant factors and *p*-values (<0.05) are in bold.

Elytron Dimension	Sex	*E. livingstoniana*		*E. sandwicensis*		Wilcoxon Test (One-Sided)		
		*n*	Mean ± SE (mm)	*n*	Mean ± SE (mm)		*Z*	*p*
Width	female	10	1.27 ± 0.025	10	1.30 ± 0.038	**plant**	1.82	**0.** **034**
	male	10	1.23 ± 0.025	10	1.29 ± 0.027	sex	−0.95	0.170
Length	female	10	2.51 ± 0.046	10	2.54 ± 0.068	plant	1.47	0.071
	male	10	2.45 ± 0.030	10	2.49 ± 0.049	**sex**	−1.77	**0.** **038**
Area (estimated)	female	10	3.19 ± 0.11	10	3.33 ± 0.17	**plant**	1.84	**0.** **033**
	male	10	3.02 ± 0.09	10	3.22 ± 0.12	sex	−1.35	0.088
Width/length	female	10	0.505 ± 0.0042	10	0.514 ± 0.0086	plant	1.72	0.085
	male	10	0.501 ± 0.0068	10	0.520 ± 0.0074	sex	0.18	0.430

## Data Availability

The data presented in this study are available on reasonable request from the corresponding authors.

## Abbreviations

The following abbreviations are used in this manuscript:

SI	*Specularius impressithorax*

## Appendix A

**Table A1:** Compiled host plant association of *Specularius impressithorax* from native and introduced regions.

Species	Native Regions	Field Infestation	References
*Erythrina abyssinica* Lam. ex DC.	Eritrea, Ethiopia, Kenya, Republic of the Congo, Rhodesia, South Africa, Sudan, Tanzania, Mozambique, Nyasaland, Uganda, Zaire, Zimbabwe	Field	[25,55,56]
*Erythrina americana* Mill.	Mexico, C. America, Belize, Guatemala, southern N. America	Field, Hawaii 2006	[22,23,57]
*Erythrina berteroana* Urb.	Central America, Colombia, Guatemala, Mexico, Venezuela	Field, Hawaii 2017, Honduras 2024	[58]
*Erythrina caffra* Thunb.	Mozambique, South Africa	Field	[51]
*Erythrina coralloides* DC.	Mexico, and Central America	Field	[59]
*Erythrina crista-galli* L.	Argentina, Bolivia, Brazil, Paraguay, United States of America, Uruguay	Field, Hawaii 2006	[22]
*Erythrina eudiphylla* Hassk. ex Backh	Indonesia	Field, Hawaii 2006	[22,23]
*Erythrina folkersii* Krukoff & Moldenke	Belize, Guatemala, Honduras, Mexico Gulf, Mexico Southeast, Mexico Southwest	Field, Kaluakauila, Coll. Mexico	[52]
*Erythrina humeana* Spreng.	South Africa, Mozambique	Field, Mozambique 2007	[12,22,52]
*Erythrina latissima* E.Mey.	South Africa	Field	[52]
*Erythrina livingstoniana* Baker	East Africa, Zimbabwe, Mozambique, Malawi, Tanzania, Zambia	Field, Hawaii 2020	This study
*Erythrina lysistemon* Hutch.	South Africa, Mozambique	Field, Mozambique 2007	[22,52]
*Erythrina microcarpa* Koord. & Valeton	Jawa, Philippines	Field, Hawaii 2006	[22,23]
*Erythrina orophila* Ghesq.	Burundi, Democratic Republic of Congo, Zaïre		[12,59]
*Erythrina pallida* Britton & Rose	Tobago, Trinidad, Venezuela, Venezuelan Antilles	Field	[59]
*Erythrina sacleuxii* Hua	Kenya, Tanzania	Field	[52]
*Erythrina sandwicensis* O.Deg.	Hawaii	Field, Hawaii 2001	[22,52]
*Erythrina senegalensis* DC.	Angola, Cameroon, Chad, Ghana, Nigeria, West Africa, Mauritania	Field	[12]
*Erythrina sykesii* Barneby & Krukoff	(as hybrid: *sykesii* × *variegata*) native to Australia	Field, Hawaii 2006	[27]
*Erythrina variegata* L.	Africa, Australia, coast of India, east Asia, India, Malaysia, Oceania	Field, Hawaii 2006	[12,22,25,52]
*Strophostyles sarmentosa* Elliott	United States of America	Field	[26]
*Physostigmana mesoponticum* Taub.	Angola	Field	[27]
